# Rare presentation of immunoglobulin G4-related disease as tracheal stenosis: a case report and review of the literature

**DOI:** 10.1007/s10067-025-07541-6

**Published:** 2025-06-26

**Authors:** Emine Büşra Ata, Emre Tekgöz, Seda Çolak, Ebru Karaçalı, Fatih Mehmet Doğan, Muhammet Çınar, Sedat Yılmaz

**Affiliations:** Division of Rheumatology, Department of Internal Medicine, Gülhane Training and Research Hospital, University of Health Sciences Turkey, General Tevfik Sağlam Street, Keçiören, Ankara, Turkey

**Keywords:** Airway disease, IgG4-related disease, Trachea, Tracheal stenosis

## Abstract

Immunoglobulin G4-related disease (IgG4- RD) is a systemic inflammatory condition that can affect any part of the body, although airway involvements are rarely seen. Herein, we report a case of young female with tracheal involvement associated with IgG4-RD. She was a 16-year-old patient admitted to the hospital with dyspnea and treated with methylprednisolone and methotrexate. We reviewed the clinical characteristics of 18 previously reported cases of tracheal stenosis with IgG4-RD. The most common symptoms were dyspnea (63.2%), cough, and wheezing. Nine patients (47.4%) had isolated tracheal involvement, and five (26.3%) had organ involvement other than airway disease. Most of the patients (14, 73.7%) received systemic glucocorticoid therapy for remission induction, while surgical procedures (10, 52.6%) were the second preferred treatment options. No relapse was observed in any patient who received immunosuppressives and disease-modifying antirheumatic drugs (DMARDs) such as methotrexate, rituximab, azathioprine, tocilizumab, and cyclophosphamide during or after remission induction therapy. In conclusion, IgG4-RD should be kept in mind in the differential diagnosis of patients presenting with clinical findings of tracheal stenosis. This study has shown that although most IgG4-RD patients with tracheal stenosis are controlled with glucocorticoids or surgical procedures, steroid-sparing agents may be needed to prevent relapses.

## Introduction

Immunoglobulin G4-related disease (IgG4-RD) is a systemic fibroinflammatory disease that is characterized by tumefactive lesions in multiple organs such as the pancreas, retroperitoneum, salivary glands, and retroorbital region. Pathology shows abundant IgG4 (+) plasma cell infiltration and storiform fibrosis [1]. Elevated serum plasma IgG4 levels help in diagnosis. Pancreas, retroperitoneum, salivary glands, and orbital lesions are the most affected areas [2]. Although intrathoracic lesions are seen in almost half of the patients, airway stenosis, especially tracheal stenosis, is rarely seen [3, 4]. Herein, we aimed to present a case with tracheal involvement and reviewed the literature. The patient provided written informed consent for publication, and the report was conducted in adherence to the ethical principles outlined in the Declaration of Helsinki.

## Case presentation

**Fig. 1:**
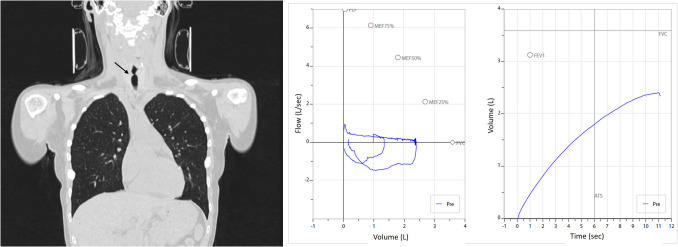
Case 1, Tracheal stenosis on thorax CT and patient’s pulmonary function test

A 16-year-old female patient admitted to hospital with shortness of breath following rhinoplasty surgery. She had no known medical history before surgery. She denied a history of hemoptysis, arthritis, hematuria, epistaxis, hearing loss, pain in the auricle, saddle nose deformity, oral aphthous ulcers, and malar erythema. There were no recurrent episodes of dyspnea and hoarseness. She had no history of tobacco or illicit drug use. Her physical examination was normal except for stridor. Her postero-anterior chest X-ray was normal. A pulmonary function test (PFT) showed forced expiratory volume in one second (FEV1) of 0.48 L (15% pred.), forced vital capacity (FVC) 2.39 L (67% pred.), and FEV1/FVC of 20%. Chest computed tomography (CT) showed stenosis in the upper part of trachea (narrowest point was 6.2 mm) (Fig. 1). Her peripheral blood cell count was normal, and biochemical examination revealed the following: C-reactive protein (CRP) 0.7 mg/L (0–5 mg/L), erythrocyte sedimentation rate (ESR) 3 mm/h (0–30 mm/h), serum IgG4 163 mg/dL (4.9–135 mg/dl), and serum IgE, 82 IU/ml (< 100 IU/ml). Anti-nuclear antibody (ANA), anti-neutrophilic cytoplasmic antibody (ANCA), rheumatoid factor (RF), and anti-citrullinated peptide (anti-CCP) were negative. Tracheal biopsy after laryngoscopy was performed, and intense fibrosis and widespread plasma cell infiltration were observed in the biopsy material obtained from the trachea. In the IgG4 specific staining, the IgG4/IgG positive cell ratio was 50%, and 80 IgG4 positive plasma cells were seen in 1 high-power field. The patient was diagnosed with IgG4-RD and prescribed methylprednisolone 24 mg/day and methotrexate 15 mg/week. The glucocorticoid dose was tapered every week. Currently, her clinical condition is stable with methotrexate 15 mg/week and methylprednisolone 4 mg/day at 10-month follow-up visit.

## Literature review

### Search strategy and selection criteria

**Fig. 2:**
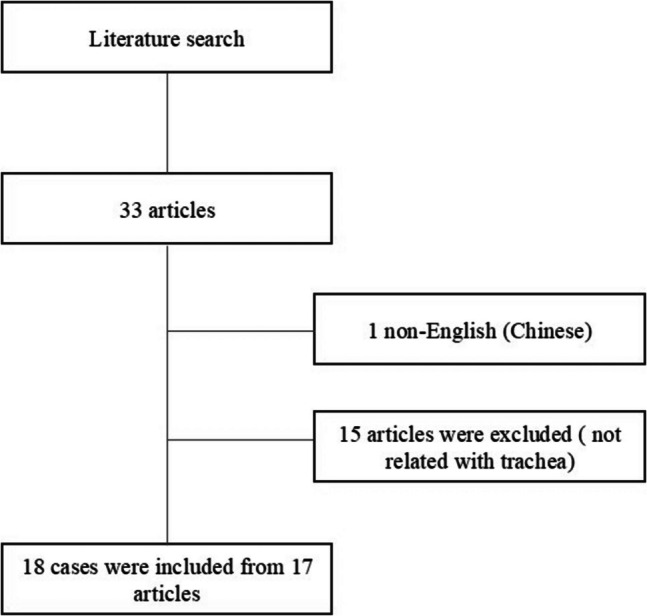
Flowchart of the study

We conducted a literature search in PubMed database (until 10 February 2025) with keywords “IgG4 related disease” and, “tracheal stenosis”, “airway disease”, and “trachea”. Irrelevant articles which do not include case presentation or clinical vignette were excluded. The references for the articles included were also reviewed. In the initial review, 33 articles were reviewed. One article was excluded because it was in Chinese, and 15 articles were excluded because they were related other parts of the airway such as larynx, pharynx, or bronchi, not to trachea. At the end 18 cases of 17 articles were included in the study (Fig. 2).

### Study population

All 18 available cases were retrieved from 17 articles and our case included in this review (Table 1). The patient characteristics, clinical presentation, site of the disease involvements, serum IgG4 level, pathological examination, therapeutic interventions, and outcomes were recorded.

**Table 1:** Clinical characteristics and treatment of IgG4-RD patients with tracheal stenosis

Number of cases	Authors	Age and gender	Presenting symptoms	Airway involvement	Other IgG4-related involvement	Biopsy	Serum IgG4 level	Treatment	Relapse	Follow-up	Outcome and complications	Radiological finding
1	Our case	16, F	Dyspnea	Trachea	None	IgG4 secreting plasma cells Fibrosis	High	Glucocorticoid, Methotrexate	None	10 months	Relieving of symptoms	Improvement of stenosis (6.2 to 8.5 mm)
2	Roncati et al.[5]	67, M	Dyspnea	Trachea	None	IgG4 secreting plasma cells Fibrosis	Normal	Surgical resection	Unknown	None	Unknown	Unknown
3	Gabrovska et al. [6]	17, F	Dyspnea, noisy and weakened breathing	Trachea	None	IgG4 secreting plasma cells	High	Glucocorticoid, partial surgical resection	None	2 years	Iatrogenic cushing	Stable tracheal stenosis (4,4 mm)
4	Oliveri Aruete et al.[7]	28, F	Dyspnea, cough, fever, epistaxis, dysphagia, and odynophagia	Trachea	None	IgG4 secreting plasma cells Fibrosis	Unknown	Surgical resection, glucocorticoid, Y-shaped endobronchial tracheal prosthesis	None	5 months	Relieving of symptoms	Significant improvement of stenosis
5	Ito et al.[8]	63, F	Cough/loss of appetite and pale stool	Trachea and main bronchi	Pancreas, submandibular gland, hilar and mediastinal lymph node	IgG4 secreting plasma cells	High	Glucocorticoid	Unknown	None	Relieving of symptoms	Significant improvement of stenosis
6	Ji et al.[9]	55, M	Cough, dyspnea	Trachea, nasal septum	None	IgG4 secreting plasma cells Fibrosis	High	Glucocorticoid	None	6 months	Relieving of symptoms	Significant improvement of stenosis
7	Kobraei et al. [10]	26, F	Dyspnea, cough	Trachea	None	IgG4 secreting plasma cells Fibrosis	Unknown	Surgical resection	Unknown	None	Relieving of symptoms	Unknown
8	Casanova et al. [11]	60, F	Stridor	Trachea	None	IgG4 secreting plasma cells Fibrosis	Normal	Surgical resection, glucocorticoid	Unknown	None	Relieving of symptoms	Unknown
9	Casanova et al.[11]	85, F	Dyspnea, stridor	Trachea	None	IgG4 secreting plasma cells Fibrosis	Unknown	Surgical resection, glucocorticoid	Unknown	None	Relieving of symptoms	Unknown
10	Wheeler et al. [12]	46, F	Dyspnea, cough, and stridor	Trachea	None	IgG4 secreting plasma cells	Normal	CO2 laser incision, steroid injection, surgical resection, rituximab	Yes (2 times) (after steroid injection and surgical resection )	3 years	Relieving of symptoms	Significant improvement of stenosis (10 mm)
11	Virk et al.[13]	22, F	Dyspnea, wheezing	Trachea, supraglottis, larynx	None	IgG4 secreting plasma cells Fibrosis	Unknown	Surgical resection	Unknown	None	Relieving of symptoms	Unknown
12	Yamashita et al. [14]	70, M	Hoarseness	Trachea and main bronchi	Parotid, salivary glands, pachymeningitis, pleural nodules, vertebrae, mediastinal lymphadenopathy	IgG4 secreting plasma cells Fibrosis	High	Glucocorticoid	None	12 months	Relieving of symptoms	Significant improvement of stenosis
13	Namireddy et al. [15]	9, F	Fever, cough, and epistaxis	Trachea, bronchi	None	IgG4 secreting plasma cells	High	Glucocorticoid, tocilizumab	Unknown	None	Relieving of symptoms	Significant improvement of stenosis
14	Torii et al. [16]	74, F	Swelling of the eyelids	Trachea and main bronchi	Eyelids and parotid glands	IgG4 secreting plasma cells Fibrosis	High	Glucocorticoid	None	6 months	Relieving of symptoms	Significant improvement of stenosis
15	Wang et al. [17]	52, M	Intermittent cough, wheezing	Trachea, bronchi	None	IgG4 secreting plasma cells	High	Glucocorticoid, cyclophosphamide, azathioprine	None	6 months	Relieving of symptoms	Significant improvement of stenosis
16	Gkikas et al. [18]	54, F	Stridor	Trachea	None	IgG4 secreting plasma cells	Unknown	Cryoablation, glucocorticoid	Yes (after cryoablation)	9 months	Relieving of symptoms	Significant improvement of stenosis
17	Sekiguchi et al. [19]	44, M	Sinus congestion, wheezing, dyspnea, and cough	Trachea and bronchi	Submandibular gland, lung parenchyma, mediastinal and hilar lymph node, left kidney	IgG4 secreting plasma cells	High	Glucocorticoid	Yes (after steroid therapy)	18 months	Relieving of symptoms	Significant improvement of stenosis
18	Noh et al. [20]	70, F	Dyspnea, facial edema	Trachea	Mediastinal mass, superior vena cava	IgG4 secreting plasma cells Fibrosis	Unknown	Surgical resection	Unknown	None	Relieving of symptoms	Unknown
19	Atienza-Mateo al. [21]	30, F	Dyspnea	Trachea and subglottis	None	IgG4 secreting plasma cells	Normal	Surgical resection, glucocorticoid, rituximab	None	4 years	Relieving of symptoms	Significant improvement of stenosis

Abbreviations: *IgG4-RD* immunoglobulin, G4-related disease, *IgG4* immunoglobulin G4, *F* female, *M* male

## Results

A total of 19 cases were included in the analysis. Fourteen (73.7%) cases were female, and median age was 52 years. Most of the presenting symptoms were dyspnea (12, 63.2%), cough, and wheezing. Six (31.6%) patients had both trachea and bronchi involvement, nine (47.4%) patients had isolated trachea involvement, and five (%26.3) patients had organ involvement other than airway disease. Serum IgG4 levels were high in nine (69.2%) patients in whom serum IgG4 levels were available. Most of the patients (14, 73.7%) received systemic glucocorticoid therapy, although surgical procedures (10, 52.6%) were the second preferred treatment options. Five patients were observed to receive immunosuppressives and disease-modifying antirheumatic drugs (DMARDs). The preferred medical treatment options in these patients were methotrexate, rituximab, azathioprine, tocilizumab, and cyclophosphamide. Most patients’ symptoms improved after treatment, but some patients’ conditions remained stable, if not completely resolved. Relapses were observed in three (27.3%) of 11 patients who had follow-up information. One of these patients received systemic glucocorticoid therapy, while the other two received local ablation therapy. The longest follow-up data is available for patients who were receiving rituximab, and no relapse was observed during this period (the mean duration of administration was 42 months) (Table 1).

## Discussion

IgG4-RD is an immune-mediated fibroinflammatory condition that can affect various regions of the body. Although mostly involved regions are the pancreas, retroperitoneum, salivary glands, orbita, lymph nodes, and aorta, it can affect any organ of the body. According to this literature review, middle-aged women are more likely to suffer from tracheal stenosis, and most patients’ symptoms improve following steroid treatment. Among immunosuppressives, rituximab appears to be more effective for achieving long-term remission.

IgG4-RD is usually encountered in male patients between the ages of 50 and 70 [2]. However, females are more likely to get head and neck involvements (e.g. thyroiditis, dacryoadenitis, and sialadenitis). Lung involvements are seen at comparable rates in both sexes. [22]. In this literature review, the median age at diagnosis was found to be 52 years, and 14 (73.7%) patients were female. However, more complete research is needed to conclude that tracheal involvement is more likely in women.

Lung is one of the affected organs in IgG4-RD. Various investigations have found that lung involvement varies between 17.6 and 23.4% in IgG4-RD [22, 23]. Radiographic examinations of patients with IgG4-RD reveal lung lesions in approximately half of the patients [24]. Lung involvement can be classified as parenchymal, airway, pleural, and mediastinal. The most prevalent lung involvements are mediastinal and parenchymal, while airway and pleural involvement are comparatively rare [4]. In a study of 90 individuals with IgG4-RD, thoracic involvement was assessed in 64 patients and found in 31 of them (48.4%). The most common thoracic involvement was mediastinum in 16 (51.6%) patients, and central airway involvement was observed in four (12.9%) patients. Thoracic involvement was associated with pancreas, retroperitoneal, and lymph node involvement, while isolated thoracic involvement was seen in only four (12.9%) patients [25]. In this review, five (%26.3) patients were observed to be involved with other organs. The most affected areas were the salivary gland, mediastinal and hilar lymph nodes, and pancreas [8, 14, 16, 19].

Dyspnea, chest pain, and cough were the most frequently reported symptoms among individuals with lung involvement [4]. In this study, although the most common symptoms were dyspnea and cough, findings more specific to tracheal stenosis such as wheezing, stridor, and hoarseness were also observed. Nonspecific laboratory abnormalities in IgG4-RD may include anemia, increased ESR, and elevated CRP. Serum IgG4 levels can help in diagnosis; however, they are not elevated in all patients. In a study of patients with IgG4-RD, serum IgG4 was found to be elevated in 51% of patients [26]. High serum IgG4 levels were detected in 58 to 82% of IgG4-RD patients with lung involvement [27, 28]. This review found that 69.2% of patients had high serum IgG4 levels, which is consistent with the literature.

The most common causes of tracheal stenosis include intubation, tracheostomy, and post-surgical stenosis, but it can also occur in inflammatory rheumatic disorders [29]. The most common rheumatic diseases are granulomatosis with polyangiitis, relapsing polychondritis, and sarcoidosis [30]. The diagnosis of relapsing polychondritis is supported by the appearance of calcifications in the trachea and concurrent involvement of the nose and ear cartilages. In granulomatosis with polyangiitis, hemoptysis, purpuric rash, sinusitis, saddle nose deformity, and ANCA tests are helpful in diagnosis. Circumferential wall thickening of the trachea with posterior membrane involvement and accompanying pulmonary cavitary nodule can be visualized in imaging. Although bronchial wall thickening and bilateral hilar lymphadenopathy are observed in sarcoidosis, the symptoms related with airway stenosis are not severe [12]. In our case, there wasn’t saddle nose deformity and auricula involvement, arthritis, ocular inflammation, hematuria, proteinuria or recurrent episodes, and no calcification or posterior tracheal membrane involvement of the trachea; therefore, we thought that diagnosis of relapsing polychondritis or granulomatosis with polyangiitis was unlikely.

Pathological examinations are useful for diagnosis. In individuals with a prior diagnosis of IgG4-RD and imaging results consistent with IgG4-RD, recurrent biopsy may not be required. Increased amount of IgG4 + cells can be observed in bronchoalveolar lavage in patient with lung involvement. [3]. Histopathological examination findings compatible with IgG4-RD are dense lymphoplasmacytic infiltrates, storiform fibrosis, obliterative phlebitis, increased number of IgG4 + plasma cells, and IgG4 +/IgG + cell ratio > 40 [31]. However, studies focused on lung involvement showed that fibrosis is less common than in other regions [32]. The presence of fibrosis was noted in 11 (57.9%) of the patients included in this review.

Glucocorticoids constitute the mainstay of treatment for IgG4-RD. Many patients achieve remission after steroid therapy. Additionally, surgical treatment is an option for lesions that cause compression and stenosis. Disease modifying antirheumatic drugs such as methotrexate, azathioprine, mycophenolate mofetil, cyclophosphamide, and tacrolimus can be used for steroid sparing agents. Rituximab, which affects by depleting B cells, is used as an effective treatment option in resistant cases [2]. Although glucocorticoids and surgery could control many of the cases in this review, rituximab, methotrexate, azathioprine, cyclophosphamide, and tocilizumab were the other therapy options for individuals who needed steroid sparing agents. While glucocorticoid treatment results in complete remission for many IgG4-RD patients, discontinuing glucocorticoids may result in relapses. In a study investigating relapse in IgG4-RD patients, the relapse rate at 36 months was found to be 36.1% [33]. Although the relapse rate regarding lung involvement is unknown, radiological residual involvement was seen in 14% of patients [28]. In this review, relapses were observed in three (27.3%) of 11 patients who had follow-up information. No relapse was observed in any patient receiving DMARDs and immunosuppressives during or after remission induction therapy. The longest follow-up data are available for patients’ receiving rituximab, but it is not sufficient to say that rituximab is a better treatment option because long-term follow-up is unknown in other cases where tocilizumab, azathioprine, or cyclophosphamide therapy was used. Residual stenosis was seen in almost all patients on radiological examinations; however, all of them were clinically stable.

The study had some limitations. If dynamic airway high-resolution computed tomography was available, fixed airway stenosis could be detected in IgG4-RD and utilized to differentiate it from relapsing polychondritis with dynamic airway collapse. However, the absence of surrogate markers for granulomatous polyangiitis or relapsing polychondritis, as well as the patient’s high serum IgG4 levels, and pathological examination confirmed as IgG4-RD diagnosis. In most cases, long-term patient follow-up was inadequate for assessing therapeutic responses. Our patient is currently being followed up, but many in the literature were assessed only at the time of diagnosis. There were no relapses detected with DMARD therapy, although the number of patients was inadequate to determine which DMARD was better. Despite these shortcomings, we believe that our study helps to draw attention to IgG4-RD, which should be considered in patients presenting to the rheumatology clinic with tracheal stenosis.

## Conclusion

IgG4-RD should be considered in individuals with tracheal stenosis; early glucocorticoid treatment can induce complete remission in many patients, and surgery may be necessary in certain situations. Additionally, DMARDs and immunosuppressive drugs may be required to prevent relapses in patients who have undergone local or surgical intervention alone or in patients whose disease flares during glucocorticoid withdrawal.
